# 

**Published:** 1987-11

**Authors:** 


Established 1883

### BRISTOL MEDICO-CHIRURGICAL JOURNAL

**NOVEMBER 1987**
**Volume 102 (iv)**

Published quarterly and distributed free on request to doctors in the South West of England. Overseas subscription £12 per annum.

**Editor**
Mr M. G. Wilson
*40 Coombe Lane, Bristol BS9 2BJ*
*Tel: 0272-682320*

**Assistant Editor**
Dr P. R. Goddard

**Editorial Committee**
Mr B. P. Jones (Secretary),
*Medical Librarian, Bristol University*
Dr J. L. Burton

**Correspondents**
Dr A. E. Adam (Taunton)
Dr J. D. Davies
Dr J. Jancar
Professor D. J. Pereira Gray (Exeter)
Dr H. G. Mather
Professor G. M. Stirratt
Mr C. Teasdale (Plymouth)
Dr J. L. G. Thomson
Dr M. J. Whitfield
Professor G. K. Wilcock
Professor R. C. N. Williamson
Dr D. W. Wright

**BRISTOL MEDICO-CHIRURGICAL SOCIETY**

**President**

Dr H. M. Norman

**Secretary**
Mr R. N. Baird,
*23 Old Sneed Park, Bristol BS9 1RG*

**Treasurer**

Dr N. C. Tricks,

*The Mill, Wrington, Bristol*

**Published by**

**Hermiston Publications,**

*2 Hill Square,*

*Edinburgh EH3 9DR*

*Telephone (031) 668 3753*

**Production Manager**

June Macnab

**Typesetting by**

*Avon Communications Ltd.,*

*Avon House, Nailsea, Bristol BS19 2DJ*

**Printed by**
*Scottish County Press, Sherwood Industrial Estate,*
*Bonnyrigg, Midlothian*

What I know remains unknown, unless by me to others shown.
*Lucilius (180–102 BC)*

**NOTICE TO CONTRIBUTORS**

Contributions are invited especially from West of England authors on a variety of subjects, e.g. Original articles relating to the practice of medicine, reports on the contemporary medical scene, obituaries, historical accounts, recollections of colleagues and events worthy to be remembered and liable to be forgotten. An article of 2,000 words with 2 illustrations will occupy 2 pages and this is a suggested, though by no means an obligatory length. Articles are preferred in the form proposed by the International Committee of Medical Editors (Vancouver System)—pamphlet obtainable from Editor of BMJ., BMA. House, Tavistock Square, London, WC1H 9JR.

**BRISTOL MEDICINE**

**Bristol Medicine acknowledges with gratitude the support it receives from Pfizer Ltd.**

Abdominal aortic aneurysms, trials and tribulations, 116.
Abdominal symptoms in general practice, 33.
Academic practice or primitive physic, 42.
AIDS, the epidemic and its control, 66.
AIDS, the origin of, 69.
Ali M. H. 110.
Ali S. 84.
Amputations—the good, the bad and the ugly, 115.
Amyloid, muco-cutaneous manifestations of 113.
Anderson J. B. 51
Appleton G. V. N. 109.
Axillary aneurysm presenting as brachial plexus palsy, 74.

Baily R. A. J. 115.
Barnes I. 52.
Bathurst N. 109.
Baxter A. S. 21.
Benjamin I. S. 108.
Bladder, use of tumour marker in carcinoma of, 52.
Bliss B. P. 20
Bloom S. R. 112.
Bogus Allergy treatment, 9.
Bond P. R. 33.
Book Reviews—
Textbook of Dermatology. Wilkinson et al., 54.
Diagnostic imaging of the chest. Goddard, 54.
The blind watchmaker. Dawkins, 54.
The Penguin Dictionary of curious and interesting words, 78.
Atlas of the surgery and management of intestinal stomas, Celestin, 80.
Heinemann Medical Dictionary, 81.
Oxford Textbook of Medicine, 96.
The Oxford Companion to Medicine, 100.
Diagnostic radiology exercises, Goddard, 102.
Operative surgery and management, Keen, 94.
British Society of Gastro-enterology, Bristol, Meeting, 108.
Burns, cultured skin in the management of, 19.
Burton J. L. 79, 44, 80, 104.

Calne R. Y. 18.
Campylobacter pyloridis—a new pathogen?. 113.
Cassava (Tapioca) consumption, the dangers of, 37.
Chadwick V. S. 110.
Chapman C. 19.
Chudry F. A. 97.
Church H. 111.
Clarkson J. P. 109.
Cobby M. J. 103.
Coeliac disease, cell mediated immunity in, 109.
Colo-rectal cancer and the blood transfusion effect, 109.
Colon, blood flow of the obstructed, 17.
Comfrey ingestion followed by hepatic veno-occlusive disease, 110.
Compston J. 111.
Congenital infections, 112.
Cook P. 85.
Cooper B. 110.
Cooper M. J. 108.
Craig D. G. 42.
Crawley E. O. 111.
Crohn's disease, Cardiff Jubilee, 108.
Crohn's disease, gastro-duodenal, 17.
Crohn's disease, taste acuity, zinc status and smoking in, 110.
Cuschieri A. 108.

Daly H. M. 113.
Daneshmend T. K. 35.
David T. J. 9.
Davies J. D. 35, 110.
Deane intercondylar knee, 114.
Deep Vein thrombosis, predictive index for, 97.
Dixon P. M. 108.
Douglas S. 110.
Downes L. 110.
Duodenal diverticula, association with cholelithiasis, 109.
Dufresne C. 97.
Dunn R. B. 75.
Dysphagia, a case of, 101.

Elmes M. E. 109.
Evans W. D. 111.
Eyre-Brook A. L. 85.

Fairclough J. 114.
Ferro M. 52.
Firth A. 110.
Flail arm splint, 85.
Frenchay Hospital, redevelopment of, 12.

Gardening 56.
Gastric carcinoid presenting with haematemesis, 41.
Gastric emptying after conservative pancreatectomy, 108.
Gastritis, a sero-diagnostic test for, 110.
Gastro-intestinal bleeding in a female runner, 110.
General practice and Hospital Medicine—a new relationship, 95.
Ghoble M. N. 19.
Gingell J. C. 53.
Granulomatous Liver Disease, 110.
Griffith H. 93.

Hall M. J. 37, 73, 110.
Ham Green Hospital, changing face of, 14.
Hamilton M. 73.
Hammonds J. C. 18.
Hand injuries, use of Kirschner wires in, 85.
Hanley D. J. 19.
Harris D. 19.
Heat treatment of serum, effect on biochemical analyses, 113.
Hepato-cellular carcinoma, pedunculated, 112.
Hewer T. F. 56.
Hinchcliffe A. 52.
Hindfoot stabilisation in rheumatoid arthritis, 115.
Hip replacement, total, isoelastic cementless, 84.
Hodgkin's disease, phenytoin (epanutin) associated, 35.
Holl S. 101.

Ibrahim N. B. N. 112.
Immuno-histochemistry of extrapulmonary small cell carcinoma, 112.
Immuno-reactive metallothionein in copper loaded human liver biopsies, 109.

Jasani B. 109.
Jenkins M. 112.
Jewell D. P. 109.
Jeyasingham K. 97.
Johnson B. G. 110.
Jones G. 114.
Journey to Jabalpur, 79.

Karagianis J. A. 109.
Kay S. 108.
Kelly M. J. 15.
Keloids, 19.
Kennedy R. H. 109.
Kirkup J. 114.

Levine D. F. 110.
Ligament prostheses, 85.
Linehan I. P. 108.
Listeria genospecies in inbred mice, differences in virulence, 112.
Logan R. F. 41.

McConnell A. A. 113.
MacGowan A. P. 112.
MacKinnon J. G. 115.
McGraw M. E. 75.
MacNulty C. A. M. 113.
Maddox P. R. 17.
Mahy N. J. 109.
Mainou-Fowler T. 112.
Malpani K. 52.
Marsh C. H. 115.
Massy J. A. 51.
May P. 114.
Mayberry J. F. 108.
Meckel's Diverticulum, imaging in the diagnosis of, 108.
Medicine in the Mountains, 80.
Miller P. 114.
Mitchell J. P. 108.
Mitchell J. R. A. 6.
Morris T. J. 108.
Morrison P. 114.
Motley R. J. 108.
Mullick S. 85.
Mutagenic potential of anti-cancer drugs, 51.

National doctor morbidity survey, 93.
Newell D. G. 110.
Nile Delta, success story in, 49.
Nolan D. J. 108.

O'Boyle P. 52.
Obituary—
Professor John Clutton-Brock, 26.
Mr Anthony Palin, 27.
Dr Terence Steen, 43.
Dr Hugh James Orr-Ewing, 82.
Dr James Macrae, 82.
Oesophagectomy without thoracotomy, 18.
Oesophagus, carcinoma of, comparison of irradiation with intubation, 18.
Oesophagus, Carcinoma of, is resection worthwhile? 18.
Orthopaedics in the developing world, 85.
Orthopaedic fellows of the Royal Society, 84.
Osteoporosis, increased prevalence in chronic liver disease, 111.
Osteotomy, corrective, in lumbar ankylosing spondylitis, 115.

Pagliero K. M. 18.
Pancreatic arteriography, 109.
Pancreatitis, chronic, total pancreatectomy in the treatment of, 108.
Paris, educational visit to, 15.
Phillips M. J. 51.
Pickering B. 18.
Polak J. M. 112.
Pollock A. 15.
Postlethwaite R. 112.
Pouch, experience with, 17.
Priddle J. D. 109.
Prostatic carcinoma, use of specific antigen in, 52.
Psychological aspects of surgery for abnormal appearance, 19.
Purry N. A. 115.
Putting the boot in, 44.

Rahamin J. 18.
Reynolds I. 85.
Reed P. I. 110.
Regan M. W. 114.
Retention, acute, pathophysiology of, 51.
Rhodes J. 108.
Rhys-Davies E. 108.
Richardson R. B. 97.
Rivett A. 52.
Roberts G. M. 108.
Roberts J. B. M. 52.
Rode J. 112.
Rose J. R. D. 108, 111.
Ross W. B. 109.
Russell R. C. G. 108.

St Georg Sledge unicompartmental knee arthroplasty, 115.
Salter Chondroplasty, 114.
Sanderson J. E. 41.
Scalenectomy in the treatment of thoracic outlet compression, 114.
Scrotal ultrasonography in assessment of orchitis, 120.
Seal V. 84.
Seale J. 66.
Shahi J. 41.
Sharpe M. 112.
Should every cow carry a government health warning? 6.
Simmonds A. 115.
Slaney, Sir Geoffrey, 116.
Smith J. M. A. 69.
Smith P. J. B. 51, 52.
Smith P. M. 111.
South West Orthopaedic Club, Meeting at Hereford, 84.
Meeting at Bath, 114.
South West Urologists Club, Meeting at Taunton, 51.
South West Surgical Club, Meeting at Plymouth, 17.
Smoking delays the onset of ulcerative colitis, 108.
Spermatogenesis, impaired in testes at risk of torsion, 51.
Spina bifida child, long term results of hip surgery in, 114.
Spinal fusion with screw fixation, 84.
Springall D. R. 112.
Staddon G. E. 97.
Stewart I. 108.
Stoddart P. G. P. 101.
Stone bashing on Dartmoor, 18.
Stower M. J. 53.
Streptokinease therapy in acute arterial occlusion, 20.
Stress incontinence, teflon injection in, 52.
Sulphasalazine, adverse reaction to, 73.

Ten years on 75.
Thomas E. A. 103.
Thomas W. E. G. 51.
Thomson M. H. 109.
Thomson W. H. F. 17.
Toboggan or not toboggan, 103.
Torrance H. B. 108.
Transplantation, whither? 18.
Troughton A. H. 103.
T. U. R. reaction, predisposing factors, 19.

Vesey S. 52.
Vesico-ureteric reflux, endoscopic teflon injection for, 53.
Virjee J. 109.

Weisl H. 114.
Wessex Branch of Association of Clinical Pathologists, Bath Meeting, 112.
Weston C. F. M. 110.
Whithead P. 97.
Williams A. J. K. 73, 110.
Williams G. 108.
Williamson R. C. N. 18, 51, 108, 109.
Wilson M. G. 80.
Wishful thinking 104.
Woodcarving 21.
Wright D. W. 14.

Yeoman P. 114, 115.
Young S. K. 115.

Zorab J. S. M. 112.

